# Gene Expression in the Salivary Gland of *Rhipicephalus (Boophilus) microplus* Fed on Tick-Susceptible and Tick-Resistant Hosts

**DOI:** 10.3389/fcimb.2019.00477

**Published:** 2020-01-21

**Authors:** Poliana Fernanda Giachetto, Rodrigo Casquero Cunha, Antônio Nhani, Marcos Valerio Garcia, Jesus Aparecido Ferro, Renato Andreotti

**Affiliations:** ^1^Embrapa Agricultural Informatics, Campinas, Brazil; ^2^Bolsista do CNPq (157460/2018-5), Programa de Pós-Graduação em Biotecnologia, Universidade Federal de Pelotas, Pelotas, Brazil; ^3^Embrapa Beef Cattle, Campo Grande, Brazil; ^4^Department of Technology, São Paulo State University, Jaboticabal, Brazil

**Keywords:** RNA-Seq, cattle tick, sialome, transcriptome, host-parasite interaction

## Abstract

The success of cattle tick fixation largely depends on the secretion of substances that alter the immune response of the host. The majority of these substances are expressed by the parasite salivary gland and secreted in tick saliva. It is known that hosts can mount immune responses against ticks and bovine European breeds, and bovine industrial crossbreeds are more susceptible to infestations than are *Bos indicus* cattle. To identify candidates for the development of novel control strategies for the cattle tick *Rhipicephalus (Boophilus) microplus*, a salivary gland transcriptome analysis of engorged females fed on susceptible or resistant hosts was performed. Using RNA-Seq, transcriptomes were *de novo* assembled and produced a total of 235,451 contigs with 93.3% transcriptome completeness. Differential expression analysis identified 137 sequences as differentially expressed genes (DEGs) between ticks raised on tick-susceptible or tick-resistant cattle. DEGs predicted to be secreted proteins include innexins, which are transmembrane proteins that form gap junction channels; the transporters Na^+^/dicarboxylate, Na^+^/tricarboxylate, and phosphate transporter and a putative monocarboxylate transporter; a phosphoinositol 4-phosphate adaptor protein; a cysteine-rich protein containing a trypsin inhibitor-like (TIL) domain; a putative defense protein 3 containing a reeler domain; and an F-actin-uncapping protein LRRC16A with a CARMIL_C domain; these genes were upregulated in ticks fed on tick-susceptible cattle. DEGs predicted to be non-secreted proteins included a small heat shock protein and the negative elongation factor B-like, both acting in a coordinated manner to increase *HSP* transcript levels in the salivary glands of the ticks fed on tick-susceptible cattle; the 26S protease regulatory subunit 6B and another chaperone with similarity to calnexin, also upregulated in ticks fed on tick-susceptible cattle; an EF-hand calcium binding protein and a serine carboxypeptidase (*SCP*), both involved in the blood coagulation cascade and upregulated in ticks fed on tick-susceptible cattle; and two ribosomal proteins, the 60S acidic ribosomal protein P2 and the 60S ribosomal protein L19. These results help to characterize cattle tick salivary gland gene expression in tick-susceptible and tick-resistant hosts and suggest new putative targets for the control of tick infestations, as those genes involved in the mechanism of stress response during blood feeding.

## Introduction

The cattle tick *R*. (*B*.) *microplus* limits the development of the cattle industry worldwide, causing production losses estimated at US $3.24 billion annually in Brazil alone (Grisi et al., 2014). The losses caused by ticks are caused primarily by their feeding in the host and by pathogens transmitted via saliva thereafter. To feed, the tick must attach to the skin of the cattle, introducing their hypostome. The success of the fixation of the tick depends on the secretion of cement substances and anticoagulants, which alter the immune response in the place of the bite but can also cause systemic effects (Mans and Neitz, 2004). In addition, the success of pathogen transmission depends on some tick molecules associated with this event (Ramamoorthi et al., 2005; Hovius et al., 2008). The majority of these substances are expressed by the salivary gland and may be secreted in the saliva.

The tick saliva contains a rich variety of pharmacologically bioactive molecules that support blood feeding. During coevolution, blood sucking ticks have adapted mechanisms to evade host detection and prevent blood coagulation by synthesizing an extensive array of molecules with anesthetic, immunosuppressive, vasodilatory, profibrinolytic, and anticoagulant properties (Mans and Neitz, 2004). Blood feeding triggers a heat shock response by arthropods, as demonstrated by the increased production of heat shock proteins in response to the increase in temperature and other stresses observed during blood meal by ticks, which has been considered a stressful event in multiple forms (Shahein et al., 2010; Benoit et al., 2011).

Gene transcripts, such as glutathione S-transferase and gamma-glutamyl transferase, can be found in salivary glands because they have physiological functions; one of these genes plays a central role in the detoxication of xenobiotic compounds (de Lima et al., 2002), such as insecticides (Nandi et al., 2015; Hernandez et al., 2018), and another of which is involved in the cross-cell membrane trafficking of amino acids and peptides and in glutathione metabolism, respectively (Mulenga and Erikson, 2011).

Many other transcripts that can code for non-secreted or secreted proteins with different physiological functions may be present in tick salivary glands. Examples of predicted non-secreted proteins are the following: calnexin, which plays a role in the quality control and assembly of proteins and glycoproteins in the endoplasmic reticulum (Williams, 2006); longistatin, which modulates biochemical reactions within the cell as the inflammatory response and has a role in anticoagulant action (Anisuzzaman et al., 2012); serine carboxypeptidase, implied to be involved in degrading hemoglobin to peptides and regulating the interaction with the host; β-N-acetyl hexosaminidases, which participates in the turnover of the chitin exoskeleton (Hogenkamp et al., 2008); leucine aminopeptidase, which belongs to a diverse group of the M17 family of Zn-metalloproteases (Maggioli et al., 2018), playing important roles in the host immune response, tick-tissue development, and pathogen transmission (Ali et al., 2015); ribosomal proteins, playing essential roles in cell growth and proliferation (Trainor and Merrill, 2014); phosphorylase kinase, a holoenzyme that activates glycogen phosphorylase (Brushia and Walsh, 1999); E3 ligase, promoting cullin neddylation, required for the regulation of NF-κB, which is crucial in immune response and apoptotic pathways (Cajee et al., 2012); and mucins, providing lubrication and protection to the epithelium (Hollingsworth and Swanson, 2004).

Among the salivary gland transcripts predicted to code for secreted proteins, the following genes may be observed: innexins, transmembrane proteins (Richards et al., 2015) that form gap junction channels and hemichannels in invertebrates, including arthropods (Güiza et al., 2018); Na^+^/dicarboxylate, Na^+^/tricarboxylate and phosphate transporter, which may play a role in osmoregulation with ion transport function (Hui et al., 2014); the phosphoinositol 4-phosphate adaptor protein, a component of a molecule complex that recruits proteins to the cell membrane (Choudhury et al., 2005), a transduction pathway in tick salivary glands (McSwain et al., 1989); monocarboxylate transporters that catalyze rapid transport of many monocarboxylates (Lew-Tabor et al., 2011), cysteine-rich proteins containing trypsin inhibitor-like (TIL) domain, belonging to the family that comprises chymotrypsin, elastase and trypsin inhibitors (Sasaki et al., 2008), which has been found ubiquitously in blood-feeding insect and tick sialomes (Karim and Adamson, 2012; Maruyama et al., 2017); BmSI-7, a trypsin inhibitor-like cysteine-rich domain family, which is involved in the inflammatory response and in injury caused by tick fixation on the bovines (Sasaki et al., 2008); defense proteins, such as defense protein 3 (Zhao et al., 2019), and ixodidin, a antimicrobial peptide (Fogaça et al., 2006), which are probably involved in the immune response in the tick; PIXR, a protein with a reeler domain, favoring the colonization of Borrelia burgdorferi in Ixodes scapularis gut (Narasimhan et al., 2017); and proteins involved in actin metabolism, such as the F-actin-uncapping protein LRRC16A isoform X2.

All of these compounds may play a role interacting on the interface of the tick-host relation and are crucial to the success of tick fixation and/or pathogen transmission. On this basis, knowledge of the transcripts that have been upregulated or downregulated in both tick-susceptible and tick-resistant cattle could guide the development of more consistent immunogens against ticks at the time when the use of vaccines has presented results with varied effects, and the type of antigen used interferes with the efficacy of the vaccine (Csordas et al., 2018). In the absence of a vaccine with these characteristics, only chemical control remains for cattle producers seeking to prevent tick infestations. In addition, the use of acaricides at higher concentrations and in association to overcome the resistance of the tick to the acaricides indicates a global crisis in tick control (Higa et al., 2019), and vaccine development is a promising alternative.

It has been known for many years that hosts can mount immune responses against ticks, and non-natural hosts can mount highly effective anti-tick immune responses, preventing successful tick fixation and halting the tick life cycle (Trager, 1939). In this way, bovine European breeds (*Bos taurus*) and their industrial crossbreeds are more susceptible to tick infestations than *B. indicus*. This resistance is associated with the immune systems of the hosts, since in the first infestation, the number of ticks completing the cycle is similar in all races (Hewetson, 1972; Mattioli et al., 1993; Ghosh et al., 1999), and with the absence of transcripts of genes encoding enzymes producing volatile compounds, which may render the host less attractive to larvae (Franzin et al., 2017). Franzin et al. (2017) concluded that resistant hosts expose ticks to an earlier inflammatory response, which in ticks is associated with significantly lower expression of genes encoding salivary proteins that suppress host immunity, inflammation and coagulation. It means, at the same time, different levels of host immunity may affect the composition of tick saliva, contributing to these outcomes. The demonstration that immunosuppressive drugs eliminate this resistance reaffirmed the immune nature of this response (Bergman et al., 2000).

In this context, we aimed to differentiate the profile of salivary gland transcripts in ticks feeding on tick-resistant and tick-susceptible bovines to identify transcripts of genes encoding proteins with the potential to be used as immunogens for tick control.

## Materials and Methods

### Ethics Statement

All experimental procedures were approved by Embrapa Beef Cattle's Ethics Committee on Animal Use according to Protocol 008/2014 and according to the requirements of the National Council for the Control of Animal Experimentation.

### Cattle, Ticks, and Sample Collection

The trial for sample collection was conducted in Campo Grande, Mato Grosso do Sul, Brazil (20° 27′ S and 54° 37′ W, altitude of 530 m), at Embrapa Beef Cattle. The climate of this region is classified as rainy tropical savanna, characterized by irregular annual rainfall distribution, a well-defined dry period during the colder months, and a rainy period during the summer months.

Two weaned male cattle aged ~8 months from two bovine genotypes were used: a tick-susceptible animal, Holstein (*Bos taurus taurus*), and a tick-resistant animal, crossbreed, resulting from the crossing of the previous susceptible breed with a tick-resistant breed, Nelore (*Bos taurus indicus*). The animals were kept in a *Brachiaria decumbens* pasture for a 4 months period for disinfestation and immunological memory loss, and they were later taken to individual stalls, where they received feed of sorghum silage (17 kg/animal/day), concentrate (1 kg/animal/day), and water *ad libitum* throughout the experimental period.

Ticks used in the experiment for artificial infestation were from a colony of *R*. (*B*.) *microplus* reared in the Laboratory of Tick Biology of Embrapa Beef Cattle. Engorged females from a stabled bovine were collected (21 days) placed in Petri dishes and incubated in a biological oxygen demand chamber (BOD) at a temperature of ~28°C and humidity of ~80%. After egg laying, eggs were collected and separated into 500-mg samples containing an equivalent of 10,000 larvae. Samples were placed in 10 ml syringes with the tips removed, sealed with cotton, and then placed again on BOD for egg incubation until larval hatching. Syringes containing non-fed larvae were used for artificial infestation of cattle. An infestation with 10,000 active larvae (12 days after hatching) was performed on the dorsum in each of the experimental animals—the tick-susceptible and tick-resistant cattle, which remained immobilized for a period of 3 h to avoid self-cleaning.

### RNA Isolation and RNA-Seq

Semi-engorged adult salivary gland samples were dissected on ice and processed immediately. Three salivary gland samples were obtained for each of the bovine genotypes. In a total of six samples, each sample contained salivary glands collected from ~10 ticks, which were used for RNA isolation following the TRIzol^®^ reagent protocol (Life Technologies, Carlsbad, CA). The purity and amount of total RNA were assessed by electrophoresis in a 1.2% agarose gel stained with ethidium bromide and by spectrophotometry at 260 and 280 nm in a Nanodrop ND-1000 spectrophotometer (Thermo Fisher Scientific Inc., MA, USA). Total RNA quality and integrity were analyzed using an Agilent 2100 Bioanalyzer^®^ (Agilent Technologies, Palo Alto, USA). RNA was stored at −70°C until further analysis.

Library preparation and next generation sequencing using the RNA-Seq technique were conducted at the Animal Biotechnology Laboratory of Luiz de Queiroz College of Agriculture, University of São Paulo (ESALQ-USP, Piracicaba, SP), following the stranded TruSeq RNA Sample Prep Kit protocol (Illumina, Inc., San Diego, CA, USA). The cDNA libraries were sequenced in the Illumina HiSeq 2500 System (Illumina, Inc., San Diego, CA, USA), generating 2 × 150 bp paired-end reads, according to the standard manufacturer protocol.

### Bioinformatics Analysis

Data obtained by HiSeq 2500 sequencing were analyzed using the Real Time Analysis (Illumina) software, which makes the calls from the sequencing images, converting them into a FASTQ format. Evaluation of the sequencing reads was performed using FastQC v0.11.4 (http://www.bioinformatics.babraham.ac.uk/projects/fastqc/). Read sequences were subjected to adapter trimming and quality filtering using Trimmomatic (v0.35) (Bolger et al., 2014) with default parameters, except for a head crop of 15 bases and a read minimal length of 30 bases. Reads were also checked for foreign RNA contamination. A similarity search against the NR database (v04/18) using either blastx (v2.6.0) or DIAMOND (v0.9.17) (Buchfink et al., 2015) was performed to identify and filter hits to mammal and bacterial sequences above 50% identity and e-value ≤ 1e-20.

Trinity software (v2.6.6) (Grabherr et al., 2011) was used to *de novo* assembly of a reference transcriptome using all filtered samples, as described in https://github.com/trinityrnaseq/trinityrnaseq/wiki. Inchworm kmer coverage was set to 3, and contig minimal length was set to 300 bases. Assembly quality was accessed through mapping back each library to the *R*. (*B*.) *microplus* salivary gland reference transcriptome using Bowtie2 (v2.3.4) (Langmead and Salzberg, 2012) and Trinity scripts. Mapped contig sequences were recovered for each genotype to compose the transcriptomes.

The completeness of the *R*. (*B*.) *microplus* salivary gland transcriptome was evaluated using BUSCO (Benchmarking Universal Single-Copy Orthologs) by comparing the transcriptome against a set of highly conserved single-copy orthologs of the known ancestral Arthropoda proteins (arthropoda_odb9, creation date: 2017-02-07, number of species: 60, number of BUSCOs: 1066) (Simão et al., 2015).

Differentially expressed genes (DEGs) between ticks fed on susceptible cattle and ticks fed on resistant cattle were identified by Trinity RSEM/edgeR differential expression analysis with FDR (False Discovery Rate) <0.05 and Fold Change ≥2. A preliminary similarity search, as described previously, was also performed in the DEG set of transcripts.

TransDecoder (v 5.0.0) (Haas et al., 2013) was used to obtain peptide ORFs from differentially expressed genes. Potential signal peptides and transmembrane domains were predicted from ORFs to identify secreted proteins according to the Min (2010) animal pipeline, which consists of filtering sequences that pass through Phobius (v 1.01), WolfPsort (v 0.02) TMHMM (v 2.0c) TargetP, and PS_SCAN tools.

To further investigate the biological functions of the DEGs, they were annotated to the Gene Ontology (GO) database for biological process (BP), molecular function (MF), and cellular component (CC) and to KEGG (Kyoto Encyclopedia of Genes and Genomes) for biological pathways using Blast2GO PRO (https://www.blast2go.com/). DEGs from the most represented GO terms and KEGG pathways were discussed further.

## Results

### Gene Expression Profile and Secreted Proteins of Semi-engorged *R*. (*B*.) *microplus* Salivary Gland Fed on Tick-Susceptible and Tick-Resistant Cattle

**Figure 1:**
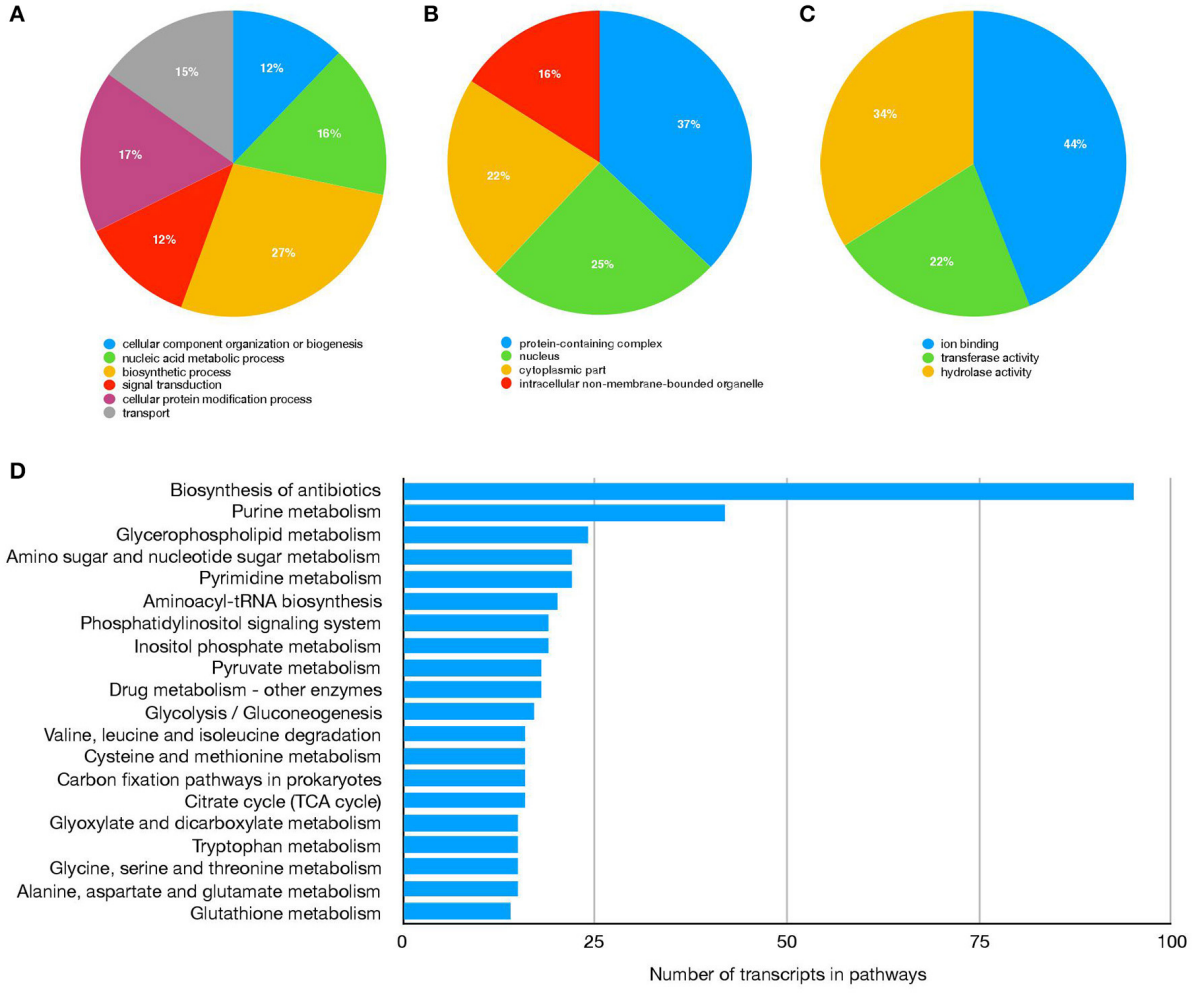
*R*. (*B*.) *microplus* salivar gland transcriptome functional annotation. Transcripts distribution based on GO categories Biological process **(A)**, Cellular component **(B)**, and Molecular function **(C)**. Top 20 processes in KEGG pathways **(D)**.

Sialotranscriptomes from *R*. (*B*.) *microplus* fed on tick-susceptible (Holstein) and tick-resistant (Holstein × Nelore crossbreed) cattle were generated using RNA-Seq technology and *de novo* assembly. A total of 74,639,552 reads were obtained for ticks fed on Holstein cattle, and 63,013,658 reads were obtained for ticks fed on crossbreed cattle. Reads post-processing with Trimommatic resulted in a 99.9% recovery for the Holstein and crossbreed reads dataset, of which 92.53 and 92.79%, respectively, were realigned to the assembly with bowtie2. Trinity produced a total of 235,451 contigs with a mean length of 954 nt and a N50 of 1,624 nt. Completeness of the assembly, as evaluated using BUSCO, revealed that 93.3% of conserved genes across Arthropoda were present (994 out of 1,066). Complete and single-copy genes found (C) were 24.4%, complete and duplicated genes found (D) were 68.9%, fragmented genes found (F) were 5.0%, and genes missing (M) were 1.7%. To identify the function of the transcriptome-predicted genes, all 235,451 contigs were analyzed using Blast2GO PRO software, returning a total of 71,757 annotated transcripts with a sequence identity ≥50% and e-value ≤1e−10 (Table 1, Supplementary Material). Gene Ontology and KEGG categorizations of the annotated transcriptome are shown in Figure 1.

**Table 1:** Putative secreted proteins^a^ transcribed in *R*. (*B*.) *microplus* salivary glands.

**Putative secreted protein**	**e-value^b^**	**Similarity^c^ (%)**	**LogFC**	**FDR**	**Taxonomy name^d^**
Na^+^/dicarboxylate, Na^+^/tricarboxylate, and phosphate transporter, putative	0.0E0	82.81	0.96	2.3e−02	*Ixodes scapularis*
Beta-N-acetylhexosaminidase, putative	1.9E−160	67.95	2.34	2.4e−03	*Ixodes scapularis*
TPA_inf: hypothetical protein 384	3.5E−15	63.22	4.32	1.8e−02	*Amblyomma variegatum*
Acid methyltransferase, putative	1.1E−52	68.66	2.78	4.4e−02	*Ixodes scapularis*
Phosphoinositol 4-phosphate adaptor protein, putative	8.0E−71	78.47	−1.50	2e−02	*Ixodes scapularis*
Innexin, putative	4.7E−156	80.86	−3.35	4.8e−03	*Ixodes scapularis*
Monocarboxylate transporter 10-like	7.4E−122	68.14	1.37	2.4e−02	*Parasteatoda tepidariorum*
Secreted protein, putative	1.4E−8	56.06	4.13	4.2e−02	*Ixodes scapularis*
Putative defense protein 3	1.2E−14	49.98	1.80	4.5e−02	*Crassostrea gigas*
F-actin-uncapping protein LRRC16A isoform X2	0.0E0	3.44	−0.83	2e−03	*Limulus polyphemus*

a *Putative secreted proteins predicted by Min (2010) pipeline, using Phobius, WolfPsort, TMHMM, TargetP, and PS_SCAN*.

b *e-value from Blast result*.

c *Blast mean similarity*.

d *Blast hit taxonomy name. Log FC with negative signal means that transcripts are upregulated in ticks fed on resistant cattle*.

TransDecoder translation resulted in a total of 1,815 predicted ORFs, which were used for secreted protein identification. According to the pipeline proposed by Min (2010), a total of 20 ORFs were predicted as putative secreted proteins from the salivary glands (Table 1), corresponding to 15 candidate genes.

### Differentially Expressed Genes Related to Cattle Resistance to Tick

**Figure 2:**
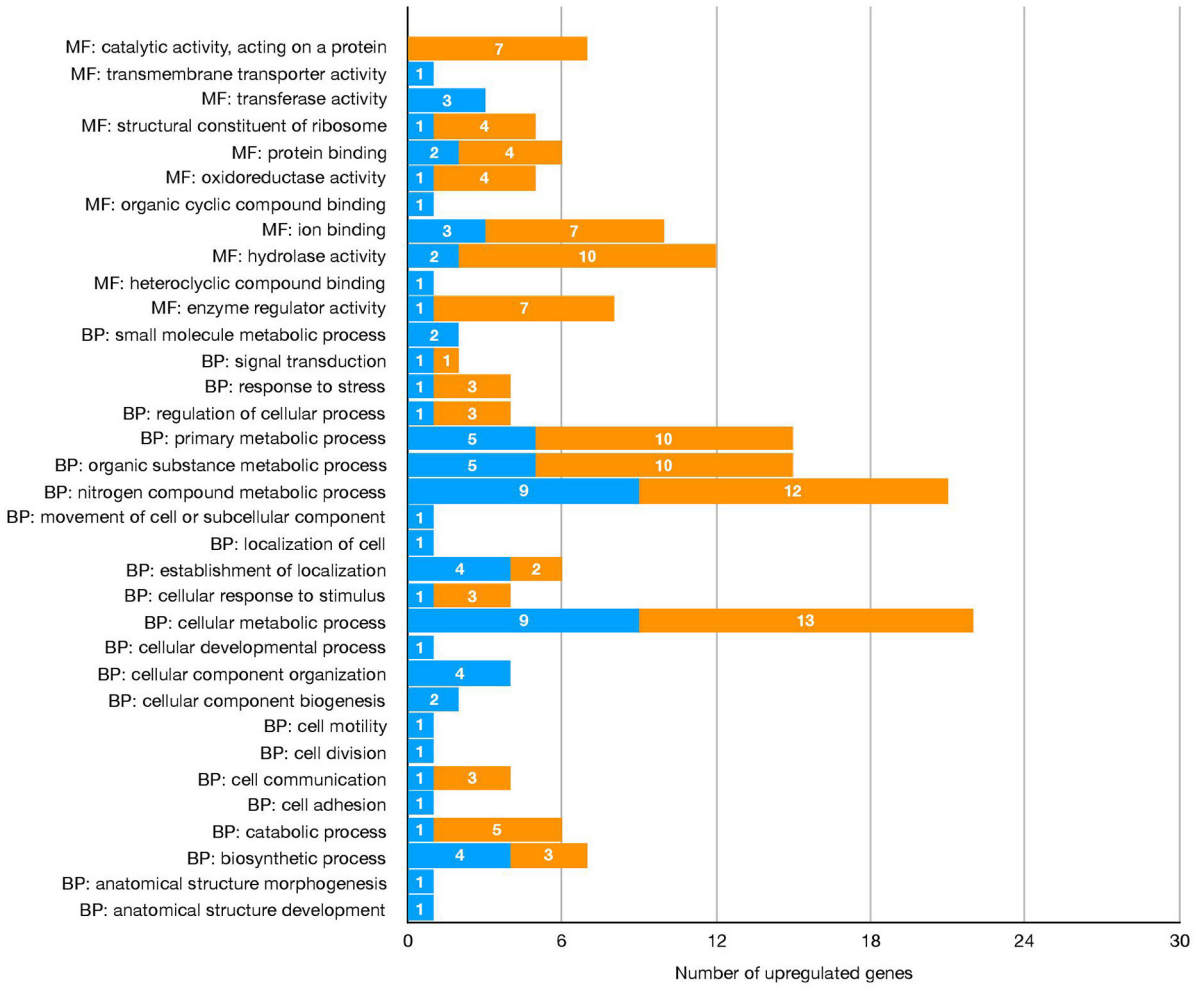
Number of the differentially expressed genes upregulated in salivary glands of *R*. (*B*.) *microplus* fed on Holstein (susceptible) and crossbreed (resistant) cattle, based on GO categories Molecular function (MF) and Biological process (BP).

The differential expression analysis identified 137 sequences as DEGs, corresponding to 126 candidate genes (Figure 1, Table 2, Supplementary Material). Gene Ontology analysis showed that the molecular functions most represented among DEGs upregulated in salivary glands from *R*. (*B*.) *microplus* fed on tick-susceptible and tick-resistant cattle were *ion binding* and *hydrolase activity*, while the most represented biological processes were the *nitrogen compound metabolic process* and the *cellular metabolic process* (Figure 2 and Table 2).

**Table 2:** Differentially expressed genes (DEG) upregulated in salivary glands from ticks fed on susceptible-tick cattle (Holstein) and ticks fed on resistant-tick cattle (Holstein × Nelore crossbreed) from most represented molecular functions and biological process GO terms.

**DEG**	**e-value^a^**	**Similarity^b^ (%)**	**FC**	**FDR**	**SP**	**Taxonomy name^c^**
**UPREGULATED IN SALIVARY GLANDS FROM TICKS FED ON HOLSTEIN CATTLE**						
Serine carboxypeptidase	4.5E−103	71.02	4.38	3.6e−05	N	*Haemaphysalis longicornis*
RNA-directed DNA polymerase from mobile element jockey-like	4.2E−126	51.45	0.88	4.7e−02	N	*Strongylocentrotus purpuratus*
Phosphorylase kinase, putative	0.0E0	81.17	0.95	4.8e−03	N	*Ixodes scapularis*
Conserved hypothetical protein	0.0	52.31	0.71	3.2e−02	Y	*Ixodes scapularis*
Neuroendocrine convertase 1	5.3E−245	83.98	1.59	3.6e−02	N	*Ixodes scapularis*
Negative elongation factor B-like	2.4E−128	80.98	1.90	1.5e−02	N	*Centruroides sculpturatus*
MAP kinase-activated protein kinase 3	1.0E−8	100.00	1.69	1.5e−03	N	*Ixodes scapularis*
Longistatin	1.5E−30	57.72	1.71	3.1e−02	N	*Haemaphysalis longicornis*
Leucine aminopeptidase	2.7E−242	90.63	3.52	1.2e−03	N	*Haemaphysalis longicornis*
Glycerol-3-phosphate dehydrogenase	6.9E−12	92.50	3.15	8e−03	N	*Ixodes scapularis*
Gamma-glutamyltransferase, putative 1^e^	6.8E−145	67.82	2.06	1.3e−02	N	*Ixodes scapularis*
Gamma-glutamyltransferase, putative 2^d^	4.7E−146	68.21	1.95	1.5e−02	N	*Ixodes scapularis*
Uncharacterized protein LOC111632264 isoform X1	6.4E−104	80.20	0.90	2.7e−02	N	*Centruroides sculpturatus*
Conserved hypothetical protein XP_002414744.1	2.5E−142	96.48	1.73	8.2e−03	N	*Ixodes scapularis*
Coagulation factor precursor putative	1.6E−53	69.47	2.74	4.1e−02	N	*Ixodes scapularis*
Calnexin	6.5E−118	92.70	2.98	2.9e−04	N	*Ixodes scapularis*
Bleomycin hydrolase	1.1E−82	41.18	1.85	1.6e−04	N	*Zootermopsis nevadensis*
Beta-N-acetylhexosaminidase, putative	1.9E−160	67.95	2.34	2.4e−03	Y	*Ixodes scapularis*
Hypothetical protein IscW_ISCW006057	5.3e−240	57.73	0.51	3e−02	N	*Ixodes scapularis*
Small heat shock protein I	4.7E−27	83.95%	3.71	1.4e−02	N	*Rhipicephalus annulatus*
Ribosomal protein P2	1.9E−24	98.48%	2.56	1e−02	N	*Haemaphysalis qinghaiensis*
RNA-directed DNA polymerase from mobile element jockey-like	4.2E−126	51.45	0.88	4.7e−02	N	*Strongylocentrotus purpuratus*
Pantothenate kinase PanK, putative	2.8E−203	92.23	1.79	4.1e−02	N	*Ixodes scapularis*
26S protease regulatory subunit 6B	3.9E−211	95.68	2.57	1.4e−02	N	*Pediculus humanus corporis*
**UPREGULATED IN SALIVARY GLANDS FROM TICKS FED ON CROSSBREED CATTLE**						
Conserved hypothetical protein	1.8E−98	87.45%	2.76	1.2e−03	N	*Ixodes scapularis*
Hypothetical protein IscW_ISCW000551	2.0E−202	50.26	0.65	2.8e−02	N	*Ixodes scapularis*
Pentatricopeptide repeat-containing protein, putative	8.7E−130	96.51	1.84	1e−02	N	*Ixodes scapularis*
Mucin-17-like isoform X3	2.0E−18	69.41	0.72	1.6e−02	N	*Limulus polyphemus*
Conserved hypothetical protein	1.9E−99	91.71%	2.13	1.3E−02	N	*Ixodes scapularis*
Fanconi anemia group I protein-like	4.9E−181	54.43%	0.58	3.2E−02	N	*Crassostrea virginica*
ATP synthase H+ transporting, mitochondrial F1 complex, delta subunit precursor	1.0E−68	94.58	3.69	4.5E−02	N	*Ixodes scapularis*
Ribosomal protein L19	2.0E−75	94.04%	3.39	4E−02	N	*Ixodes scapularis*

a *e-value from Blast result*.

b *Blast mean similarity*.

c *Blast hit taxonomy name*.

*FC, fold change; FDR, false discovery rate; SP, predicted signal peptide that target proteins to be either secreted or to be a transmembrane protein; Y—signal peptide predicted, N—signal peptide not predicted*.

d,e *Transcript isoforms*.

## Discussion

### DEGs Between Ticks Fed on Tick-Susceptible and Resistant Cattle

Blood feeding in arthropods is a stressful event in multiple forms and physiological targets. Energetic, thermal, osmotic and oxidative stresses are among the consequences of the rapid ingestion of large amounts of warm blood (Pereira et al., 2017). The large volumes of blood that arrives quickly to the midgut may increase the temperature of blood-feeding arthropods by up to 15°C in <1 min, and the thermal stress generated by the blood meal can trigger a heat shock response by arthropods, as demonstrated by the increased production of heat shock proteins (Benoit et al., 2011).

A transcript upregulated in ticks fed on tick-susceptible cattle showed similarity with the sequence of a small heat shock protein I gene cloned from salivary glands of *Rhipicephalus annulatus—*the RasHSPI (Hussein et al., 2014). The authors verified a strong immunogenic effect of the RasHSPI recombinant protein in rabbits, suggesting that it could be used as a potential protective antigen (Hussein et al., 2014). Previously, another sHSP also cloned from *R. annulatus* salivary glands, a RasHSPII, showed a role as a molecular chaperone, conferring protection at least or specifically to the tick salivary glands against the increase in temperature and other stresses observed during blood feeding by ticks (Shahein et al., 2010). Guilfoile and Packila (2004) verified an increase in the expression of a gene with similarity to a HSP70 in *I. scapularis* females and males throughout blood feeding and suggested that protein encoded by the gene may be required to stabilize tick proteins during ingestion of warm blood from the host or even when the parasite is in contact with the hot skin of the host. An increase in the expression of a small heat shock protein, HSP16, was also observed during the engorgement of *I. scapularis* females (Xu et al., 2005). According to Benoit et al. (2019), suppression of HSPs through RNA interference (RNAi) also leads to impaired digestion and subsequent egg production, indicating that prevention or repair of thermal-associated damage is critical to allow blood feeding arthropods to maximize egg production from a blood meal.

Recently, the role of an arthropod HSP70-like molecule in fibrinogenolysis during blood meal has been reported (Vora et al., 2017), as well as the protective effect of cellular protein integrity observed with the overexpression of genes from the heat shock protein family. In our results, in addition to an increase in heat shock gene expression observed in ticks fed on tick-susceptible cattle, downregulation of a transcript similar to the negative elongation factor B-like (*NELF*) was observed. Ghosh et al. (2011) found that *NELF* depletion in Drosophila, mediated by RNA interference, resulted in a delay in the dissociation of heat shock factors (HSF), the transcription factor that upregulates genes encoding heat shock proteins of the *HSP* genes during heat shock recovery. Thus, the observed increase in *HSP* transcript levels in the salivary gland of the ticks fed on tick-susceptible cattle may be related to the delay in the dissociation of the HSF from the *HSP* genes due to the downregulation of the *NELF*. This coordinated action may contribute to the success of the parasite in its interaction with the host.

Two isoforms of a transcript with similarity to an *I. scapularis* gamma-glutamyl transferase (*GGT*) were upregulated in ticks fed tick-susceptible cattle. GGT is an enzyme present in cell membranes and is involved in cross-cell-membrane trafficking of amino acids and peptides and in glutathione metabolism (Mulenga and Erikson, 2011). Glutathione S-transferases (GST) are proteins that belong to a multigene family and play a central role in the detoxification of xenobiotic compounds (de Lima et al., 2002), with increased expression levels being correlated with insecticide resistance in various organisms, including ticks (Nandi et al., 2015; Hernandez et al., 2018). GGT plays key roles in intracellular reduced glutathione (GSH) homeostasis by breaking down the extracellular molecule and providing cysteine for intracellular *de novo* synthesis of GSH (Accaoui et al., 2000; Zhang et al., 2005). Glutathione biosynthesis by GGT is important to maintain intracellular reduced glutathione and the cellular redox state. Additionally, during oxidative stress in rodents, GGT gene expression was increased, which is believed to constitute an adaptation to stress (Zhang et al., 2005). The authors also related that increased expression of GGT during oxidative stress facilitates GSH turnover, *de novo* GSH synthesis, and metabolism and detoxification of GSH conjugates, increasing cell resistance to subsequent stress. In *Haemaphysalis longicornis*, the analysis of two GSTs using real-time PCR showed that gene expression increases continuously in salivary glands and other organs of the tick as blood-feeding progresses until female engorgement, demonstrating the possible role of GSTs in coping with oxidative stress caused by blood-feeding (Hernandez et al., 2018).

The 26S proteasome, the heart of the ubiquitin-proteasome system, is a catalytic machine that cleaves most intracellular ubiquitinated proteins, maintaining cellular homeostasis by means of the recognition, unfolding, translocating and cleavage of protein substrates (Wang and Maldonado, 2006; Gallastegui and Groll, 2010). This proteasome is involved in the regulation of many crucial processes in the cell, such as the cell cycle, apoptosis, signal transduction, protein processing and immune and stress responses (Coux et al., 1996). In our study, the 26S protease regulatory subunit 6B was upregulated in salivary glands from ticks fed tick-susceptible cattle. An increase in the abundance of proteasome proteins was verified in *Trypanosoma cruzy* exposed to the antiparasitic benznidazole (Andrade et al., 2008). In the small brown planthopper *Laodelphgax striatellus*, the vector of the rice stripe virus, an increase in the accumulation of the virus was observed after disrupting the 26S proteasome, indicating that the small brown planthopper 26S proteasome plays a role in defense against rice stripe virus infection by regulating virus accumulation (Xu et al., 2015). In vertebrates, another function associated with proteasome proteolytic activity, first suggested by Yang et al. (1992), is that the proteasome is also a proteolytic generator of antigenic peptides that can be presented by class I MHC molecules. A 26S proteasome non-ATPase regulatory subunit protein was characterized as an immunogenic enzyme in 24 h fed *I. scapularis* saliva (Lewis et al., 2015), and in *H. longicornis*, the increased expression of the 26S proteasome regulatory subunit protein indicated an increase in expression of the ubiquitin-proteasome system during salivary gland degeneration in engorged females, certainly as a consequence of the programmed cell death that salivary glands undergo after tick engorgement (Wang et al., 2019).

Another chaperone, a transcript with similarity to calnexin (*CNX*), was upregulated in ticks fed tick-susceptible cattle. Calnexin is a chaperone involved in the quality control of proteins, ensuring correct folding and assembly of many glycoproteins in the endoplasmic reticulum (Williams, 2006). The endoplasmic reticulum is a specialized organelle that plays essential and central functions in the cell, including protein folding assisted by a battery of molecular chaperones. Situations that reduce the protein folding capacity of the endoplasmic reticulum, which results in the accumulation and aggregation of unfolded proteins, cause a condition known as endoplasmic reticulum stress (Guérin et al., 2008). In *Schyzosaccharomyces pombe*, calnexin overexpression is involved in the induction of apoptosis triggered by endoplasmic reticulum stress (Guérin et al., 2008). In the midgut of the argasid tick *Ornithodoros moubata* female, a calnexin protein has been identified and determined to be involved with the stress responses associated with blood digestion (Oleaga et al., 2017). A calnexin transcript was upregulated in the *Ixodes ricinus*-derived cell line IRE/CTVM19 at day 2 post-infection with the flavivirus tick-borne encephalitis virus (TBEV), which may have an important role in the response of tick cells to virus infection (Weisheit et al., 2015). In *R*. (*B*.) *microplus*, calnexin was predicted as a transmembrane protein and has been suggested as a target for tick control strategies because it showed a higher VaxiJen score (0.91) than that predicted for Bm86 (0.77) in a study from Richards et al. (2015). Calnexin has also been suggested as a promising vaccine candidate against multiple fungal pathogens because it induces fungal antigen-specific CD4^+^ T cell expansion and resistance to lethal challenge in mice with multiple fungal pathogens (Wüthrich et al., 2015).

The tick saliva contains a rich variety of pharmacologically bioactive molecules that support blood feeding. During coevolution, blood sucking ticks have adapted mechanisms to evade host detection and prevent blood coagulation by synthesizing an extensive array of molecules with anesthetic, immunosuppressive, vasodilatory, profibrinolytic, and anticoagulant properties (Mans and Neitz, 2004).

A transcript similar to longistatin, an EF-hand calcium binding protein, was overexpressed in ticks fed tick-susceptible cattle. The EF-hand calcium binding proteins modulate several crucial biochemical reactions within the cell and have a role in the blood clotting cascade, being secreted in some rare cases, as observed for the longistatin transcript identified by Anisuzzaman et al. (2012). The authors verified an overexpression of longistatin in salivary glands of the ixodide *H. longicornis* adults pre-engorged (96 h) and engorged (120 h) followed by an abrupt reduction in expression level with tick detachment, showing that longistatin clearly performs a vital function in the feeding process of the parasite through its anticoagulant action, resulting in fibrinogen hydrolysis and plasminogen activation. The post-transcriptional silencing of the longistatin gene in *H. longicornis* completely disrupted the ability of the parasite to form a blood pool and perform blood meals (Anisuzzaman et al., 2011).

In addition to the anticoagulant effect, longistatin has also been found to be able to modulate the inflammatory response of the host in a host-parasite interaction. The receptor for advanced glycation end products (RAGE) is highly expressed constitutively in different cell types of the skin and is responsible for immune activation mediation at inflammation sites. Anisuzzaman and Tsuji (2015) demonstrated that longistatin secreted in *H. longicornis* saliva binds to RAGE, modulating the host immune response and suppressing the inflammation associated with the lesion caused by the tick bite, thereby ensuring the successful acquisition of host blood. Immunization of mice with recombinant longistatin induced high levels of IgG protective antibodies against ticks, as well as reduced repletion of ticks by ~54%, post-engorgement body weight by ~11%, and nymph molting by ~34% (Anisuzzaman et al., 2012). *H. longicornis* treated with dsRNA failed to form a blood pool necessary for the proper blood meal, which led to a reduced engorged tick weight (Anisuzzaman et al., 2011).

Other binding Ca^++^-containing EF-hand motif molecules, similar to longistatin, have recently been identified from *Strongyloides venezuelensis*, the venestatin (Tsubokawa et al., 2017) and *Aedes aegypt*—the salivary factor LTRIN (Jin et al., 2018), which were upregulated promptly after larval invasion through the host's skin and during mosquito blood feeding, respectively.

A transcript similar to a serine carboxypeptidase (*SCP*) was upregulated in ticks fed on susceptible cattle compared to those fed on resistant cattle. SCPs are proteolytic enzymes that target serine in their catalytic activity and catalyze the hydrolysis of C-terminal residues in peptides and proteins (Anderson et al., 2008). In mammals, SCPs are involved in signaling events, such as the generation of bioactive peptides in the blood coagulation cascade (Turk, 2006), but little is known about SCPs in tick species. In *Ixodes ricinus*, enzymes as cathepsins B, C, and D, legumain, serine carboxypeptidase and leucine aminopeptidase were characterized as the main enzymes acting in the tick hemoglobinolytic system at midgut (Horn et al., 2009). Motobu et al. (2007) identified an *H. longicornis* SCP predominantly expressed in tick midgut upregulated in response to blood feeding, which has been described to play a role in hemoglobin degradation. SCPs were highly expressed in salivary glands and viscera from fully engorged *Ixodes holocyclus* female ticks (Rodriguez-Valle et al., 2018). The secretory/excretory proteome of helminths parasites identified SCPs that are part of a protein complex that regulates parasite-host interaction (Morassutti et al., 2012). Radulović et al. (2014) identified SCPs transcribed in the salivary gland of *Amblyoma americanum* that could act to regulate the interaction with the host.

Insect cells usually express a number of β-N-acetylhexosaminidases, which are important in various developmental stages during their life cycle, primarily in the turnover of the chitin exoskeleton (Hogenkamp et al., 2008). According to You and Fujisaki (2009), molecules associated with molt act to protect organisms against pathogen invasion, on peritrophic membrane control and other functions necessary during blood feeding and molting in ticks and may be candidates for parasite control. In our results, a DEG similar to a β-N-acetylhexosaminidase and upregulated in ticks fed on tick-susceptible cattle was found in salivary glands. Del Pino et al. (1998) inoculated anti-β-N-acetylhexosaminidase antibodies obtained against the enzyme purified from larvae of *R*. (*B*.) *microplus* into fully engorged, adult females and verified a decrease in the reproductive efficiency of *R*. (*B*.) *microplus*. Chitin is a polysaccharide that consists predominantly of unbranched chains of N-acetylglucosamine and is present together with a protein matrix in the cuticle of arthropods, including *R*. (*B*.) *microplus* (Hackman, 1975). Ticks periodically shed their old cuticles and resynthesize new ones, a process that is mediated by the elaboration of chitinase in the molting fluid that accumulates between the old cuticle and the epidermis (You and Fujisaki, 2009). Female ticks of the Ixodidae family, in general, increase their mass up to 100-fold during the 7–10 days feeding period and synthesize sufficient endocuticle to thicken their cuticle by the end of the slow phase of engorgement (Flynn and Kaufman, 2015); thus, the inhibition of enzymes involved in chitin metabolism during morphogenesis might cause serious tick damage (Del Pino et al., 1998).

A transcript similar to leucine aminopeptidase (*LAP*) was upregulated in ticks fed tick-susceptible cattle. Leucine aminopeptidases belong to a diverse group of the M17 family of Zn-metalloproteases and preferentially cleave a leucine residue at the N-terminus of the proteins and peptides (Maggioli et al., 2018). Metalloproteases, which have been found in tick saliva, salivary gland, ovary and midgut, play an important role in inflammation, immunomodulation, fibrinolysis, blood protein digestion, nociception, vitellogenesis, remodeling of extracellular matrix, and pathogen transmission (Ali et al., 2015). Leucine aminopeptidases have been identified in the midgut and salivary glands of engorged *R*. (*B*.) *microplus* (Kerlin and Hughes, 1992) and in the cytosol of cells of the midgut, salivary glands type II acini and epidermal cells from *H. longicornis* (Hatta et al., 2006). Overexpression of the midgut enzyme has been observed during blood feeding in *H. longicornis*, where the disruption of the gene encoding LAP through RNAi significantly delayed onset of egg-laying and reduced egg oviposition, suggesting that HlLAP plays a role as a blood digestive enzyme and affects tick fecundity (Hatta et al., 2007). In sheep, immunization with a leucine aminopeptidase purified from *Fasciola hepatica* resulted in high levels of animal protection against the endoparasite (Piacenza et al., 1999).

Ribosomal proteins play essential roles in cell growth and proliferation through the ribosome biogenesis process to translate mRNAs into proteins (Trainor and Merrill, 2014), and the assembly of a functional ribosome is vital for successful protein synthesis. In our study, two transcripts with similarity to ribosomal proteins were found among the DEGs in the salivary gland of *R*. (*B*.) *microplus*. The first transcript, a 60S acidic ribosomal protein P2, was upregulated in the salivary glands of ticks feeding on tick-susceptible cattle.

Phosphoproteins P1 and P2 form a complex with P0 protein to raise the eukaryotic ribosomal stalk. A number of ribosome-associated proteins were identified in female tissues from *R*. (*B*.) *microplus*, including components of the 40S and ribosomal subunits, as well as the 29S and mitochondrial ribosomal subunits (Stutzer et al., 2013), and in the salivary gland immune-proteome of *A. americanum* (Radulović et al., 2014). In the salivary gland transcriptome of *Rhipicephalus appendiculatus*, most represented pathways were *ribosome, RNA transport, protein processing in endoplasmic reticulum*, and *spliceosome* (de Castro et al., 2016). Ribosomal P proteins seem to play an important role in diseases related to infections caused by intracellular protozoan parasites. Rodríguez-Mallon et al. (2015) identified an immunogenic region of ribosomal protein P0 from *Rhipicephalus* spp. ticks and a synthetic 20 amino acid peptide from this sequence showed an efficacy of 96% as a vaccine against *Rhipicephalus sanguineus* in an immunization experiment with rabbits.

The second transcript, with similarity to 60S ribosomal protein L19 (*RPL19*), was upregulated in ticks feeding on tick-resistant cattle. Despite the essential role of the ribosome in protein synthesis, RPL19 is predicted to have an extra-protein translational function, and its overexpression in cell culture activated the cellular signaling pathway of the UPR (unfolded protein response) (Hong et al., 2014). The UPR pathway is conserved from yeast to humans (Zhang et al., 2016) and activated in response to an overloading of unfolded or misfolded peptides, and its action can either have a protective effect, with the change of protein synthesis rate decreasing peptide load into the endoplasmic reticulum or inducing cell death with apoptotic characteristics (Kim et al., 2008; Hong et al., 2014). The RPL19 gene was upregulated in ticks fed tick-resistant cattle, which indicates that cell death was the main pathway activated to salivary gland cells during host-parasite interactions. In Jurkat cells, RPL19 protein expression was decreased by heat stress, although there was no significant change in mRNA expression (Zhang et al., 2016).

Phosphorylase kinase (*PhK*) is a holoenzyme that activates glycogen phosphorylase (Brushia and Walsh, 1999) which, in turn, catalyzes the hydrolysis of glycogen to generate glucose-1-phosphate. In ticks fed on tick-susceptible cattle, an upregulation in a transcript similar to *PhK* was observed.

Genes involved in stress response include molecular chaperones, such as members of the heat shock protein gene family, as already mentioned, in addition to antioxidative enzymes (e.g., catalase, superoxide dismutases, and glutathione-S-transferases) and enzymes of carbohydrate metabolism (e.g., glycogen phosphorylase and phosphofructokinase) (Barat et al., 2016). In mammals, glycogen metabolism is profoundly affected by stress, with glycogen content in peripheral tissues decreasing during stress because of increased glycogenolysis and decreased glycogen synthesis (Van Cromphaut, 2009). To date, few studies have investigated tick glycogen metabolism. However, in a study on arthropod thermal tolerance, a reduction in glycogen content was observed in *D. melanogaster* with a sudden increase in temperature from 25 to 41.2–41.3°C with a ramping rate of 0.1°C min^−1^ (Overgaard et al., 2012). As appears to have occurred with *HSPI* and *NELF* transcripts, the upregulation of *PHKA* in ticks feeding on tick-susceptible cattle may be related to the stress condition imposed by blood feeding.

A transcript that encodes a conserved hypothetical protein (XP_002414744.1) with cullin-binding and a UBA_DCNL2 domain was upregulated in ticks feeding on tick-susceptible cattle. This protein functions as an E3 ligase, promoting cullin neddylation by binding to cullins through conserved interaction surfaces on each protein (Kurz et al., 2008). Neddylation is a post-translational modification process analogous to ubiquitination, where neddylated proteins are modified through the closest relative to ubiquitin—NEDD8 protein, which attaches to a lysine residue of the cullin scaffold proteins (Sakata et al., 2007; Rabut and Peter, 2008). Neddylation is important for a number of biological processes and is required for the regulation of a multifunctional transcription factor, NF-κB, which is crucial in immune-response and apoptotic pathways (Cajee et al., 2012). The gene upregulation verified in ticks fed on tick-susceptible cattle in our study corroborates the role of pathways related to cellular proteolysis, as already mentioned in the case of upregulation of *HSP1, GGT*, and downregulation of *NELF*, in the maintenance of the susceptible host-tick interaction.

In vertebrates, mucins are abundant in the lungs and digestive tract, where they provide lubrication and protection of the epithelium against physical damage and pathogens (Hollingsworth and Swanson, 2004). Mucins are either secreted and gel-forming or attached to the membrane by special cleavable transmembrane domains (Syed et al., 2008). A mucin-like family has been identified in insects, and its family members are distributed in salivary glands, midgut, and Malpighian tubules (Barry et al., 1999; Syed et al., 2008; Hegedus et al., 2009). In our study, a transcript similar to a mucin-17-like isoform X3 was upregulated in ticks fed on crossbreed cattle. In the hemipteran *Nilaparvata lugens*, the rice brown planthopper (BPH), a mucin-like transcript, was upregulated when the insects were transferred from a susceptible rice variety to a resistant one (Huang et al., 2017). The authors suggested that the elevated mucin production might enable BPH to cope with the stress of the defense responses or help BPH to suppress the defenses of the resistant plant (Huang et al., 2017). An upregulation in a mucin-17-like isoform X3 transcript was also observed in *Diuraphis noxia* fed on an aphid-resistant wheat plant in comparison to non-resistant plants (Sinha et al., 2016). In mice immunized with *Anopheles gambiae* midgut-bound mucin cDNA, increased mortality was observed among mosquitoes fed immunized mice compared to those fed control mice (Foy et al., 2003).

Mucin and mucin-like proteins have been found in *R*. (*B*.) *microplus* (Maruyama et al., 2017) and the sialotranscriptomes of other tick species (Karim et al., 2011; Díaz-Martín et al., 2013; Radulović et al., 2014; Tan et al., 2015; Ong et al., 2016; Antunes et al., 2018), and they may function in tick feeding by coating the chitinous feeding mouthparts or the feeding lesion (Francischetti, 2010). In 1998, an antigen purified from *R*. (*B*.) *microplus* and named BMA7, exhibiting similarity to vertebrate mucins, induced partial immunity against tick infestation in cattle (Mckenna et al., 1998).

### DEGs Predicted as Salivary Gland Secreted Proteins

Innexins are transmembrane proteins that form gap junction channels and hemichannels in invertebrates, including arthropods (Richards et al., 2015; Güiza et al., 2018). Gap junctions allow endocrine signals to be rapidly shared among adjacent cells within a tissue by mediating the direct transport of ions, small molecules, and second messengers between them (Phelan and Starich, 2001). Pharmacological inhibitors of gap junctions, such as carbenoxolone, mefloquine, and meclofenamic acids, have been cited as potential insecticides in arthropod vectors, such as *Aedes aegypti* (Calkins and Piermarini, 2015). In our study, a transcript similar to innexin was downregulated in the salivary glands of ticks fed on resistant cattle. A transcript with similarity to innexin was also expressed in the salivary glands of the female tick *A. americanum* during blood feeding (Aljamali et al., 2009) and in the proteome of the saliva of the tick *O. moubata* (Díaz-Martín et al., 2013).

The gene that encodes the predicted secreted protein Na^+^/dicarboxylate, Na^+^/tricarboxylate, and phosphate transporter, was upregulated in salivary glands of ticks fed on susceptible cattle. Analyzing transcriptome changes in *Eriocheir sinensis* larvae after desalination, Hui et al. (2014) identified transcripts of many genes involved in ion transport processes, including the Na^+^/dicarboxylate, Na^+^/tricarboxylate, and phosphate transporter, to which it was attributed a potential role in osmoregulation.

A protein predicted as secreted, the phosphoinositol 4-phosphate adaptor protein, was upregulated in ticks feeding on resistant cattle. Four phosphate-adaptor proteins 1 and 2 (FAPP1 and FAPP2) are proteins that localize to the trans-Golgi network (TGN) on nascent carriers and interact with phosphatidylinositol-4-phosphate and the small GTPase ADP-ribosylation factor (ARF) through their plekstrin homology domain (Godi et al., 2004). Phosphoinositol 4-phosphate adaptor proteins are involved in lipid transport (Oleaga et al., 2017) and control the formation and fission of post-Golgi vesicles and regulate secretory transport from the TGN to the plasma membrane, and displacement or knockdown of FAPPs inhibits cargo transfer to the plasma membrane (Godi et al., 2004). In *Ornithodoros moubata* ticks, phosphoinositol 4-phosphate adaptor protein transcripts were upregulated in the midgut after blood feeding in comparison with non-fed ticks (Oleaga et al., 2017). The phosphatidylinositol-4-phosphate, in turn, is a minor plasma membrane phospholipid component of a signal transduction pathway in tick salivary glands (McSwain et al., 1989), a key regulator of membrane transport required for the formation of transport carriers from the TGN. ARF is a Ras-related GTP-binding protein that regulates the reversible binding of cytosolic coat proteins to Golgi membranes (Donaldson and Klausner, 1994).

A putative monocarboxylate transporter was identified on the *A. americanum* tick saliva immunoproteome from fed adult females (Radulović et al., 2014) and overexpressed in *R*. (*B*.) *microplus* engorged females treated with ubiquitin-63E dsRNA (Lew-Tabor et al., 2011). Monocarboxylate transporters catalyze rapid transport across the plasma membrane of many monocarboxylates, such as lactate and pyruvate (Lew-Tabor et al., 2011). A transcript similar to a monocarboxylate transporter, predicted as a secreted protein, was upregulated in ticks fed on tick-susceptible cattle.

A secreted cysteine-rich protein containing a trypsin inhibitor-like (TIL) domain was upregulated in the salivary glands of ticks feeding on tick-susceptible cattle. This family comprises chymotrypsin, elastase and trypsin inhibitors (Sasaki et al., 2008), although many extracellular proteins also contain multiple TIL domains (Wang et al., 2015). TIL domain-containing protein family members have been found ubiquitously in blood-feeding insect and tick sialomes (Karim and Adamson, 2012; Maruyama et al., 2017), and in fungal-pathogen interactions, members of this family are frequently associated with host adaptation or specialization (Schulze-Lefert and Panstruga, 2011).

In *R*. (*B*.) *microplus*, a member of this family, known as ixodidin, was purified from the hemocytes and characterized as an antimicrobial peptide, which affected the growth of *Micrococcus luteus* and *Escherichia coli* and presented inhibitory activity (Fogaça et al., 2006). BmSI-7, a protein from *R*. (*B*.) *microplus* belonging to the trypsin inhibitor-like cysteine-rich domain family showed strong inhibitory activity toward elastase, which participates in the inflammatory response and is involved in injury caused by tick fixation on bovines (Sasaki et al., 2008). A secreted cysteine-rich protein containing the TIL domain was upregulated in the proteome of fed *Amblyomma sculptum*, induced by blood feeding, compared with non-fed ticks (Esteves et al., 2017). In *R*. (*B*.) *microplus*, TIL domain-containing proteins were also upregulated in the fully engorged female proteome in comparison with partially engorged females (Tirloni et al., 2014).

A transcript similar to the putative defense protein 3 containing a reeler domain, predicted to be a secreted protein, was upregulated in ticks feeding on tick-susceptible cattle. A putative defense protein 3 was also upregulated in the proteome of a whitefly infected by two begomoviruses, the tomato yellow leaf curl virus (TYLCV) and the papaya leaf curl China virus (PaLCuCNV), as a defense response against the viruses (Zhao et al., 2019). Defense proteins with a reeler domain have been shown to have an important role in innate immune responses in a variety of insects (Bao et al., 2011; Arp et al., 2016). In *I. scapularis*, a predicted secreted protein with a reeler domain—PIXR—was induced upon feeding and upregulated in *B. burgdorferi*-infected tick guts, favoring colonization by the bacteria (Narasimhan et al., 2017).

In our results, a transcript similar to an F-actin-uncapping protein, LRRC16A isoform X2 (*LRCC16A*) with a CARMIL_C domain, a putative secreted protein, was upregulated in salivary glands from ticks feeding on tick-resistant cattle. Actins are the major constituents of the actin cytoskeleton and are essential for cell adhesion, migration, mechanosensing, and contractility in muscle and non-muscle tissues (Simiczyjew et al., 2017; Vedula and Kashina, 2018). Actin filaments grow and shrink by addition and loss, respectively, of actin monomers at the ends of filaments (Stark et al., 2017), and such regulation affects a wide range of cell processes, including development, differentiation, immunity, and inflammation (Marcos-Ramiro et al., 2014). Actin polymerization occurs primarily through elongation at the filament barbed end, and the elongation continues until the barbed end is capped by a capping protein (CP) (Yang et al., 2005). Regulation of barbed-end capping occurs by binding of the inhibitor factors to the filament, thereby protecting it from CP, by binding to CP and inhibiting its capping activity or by uncapping (Yang et al., 2005). CARMILs are multidomain proteins that regulate the actin-binding activity of CP binding directly to it and induce a conformational change that decreases its actin-capping activity (Stark et al., 2017).

Our results help to characterize cattle tick salivary gland gene expression in both susceptible and resistant hosts and suggest new putative targets for infestation control, as those genes involved in stress response mechanism during blood feeding. A possible coordinated regulation targeting a small heat shock protein and a negative elongation factor B-like genes, the latter expressed in order to maintain heat shock gene expression increased during blood feeding; along with upregulation of a 26S proteasome subunit and calnexin, other chaperone, shed light to the role of this mechanism in maintaining tick feeding. Other interactions, as the one described, are under analysis, and may evince important new targets to vaccines development.

## Data Availability Statement

The datasets generated for this study can be found in the NCBI SRA database under accessions SAMN13636118 and SAMN13636119 from BioProject PRJNA596777 (http://www.ncbi.nlm.nih.gov/bioproject/596777).

## Ethics Statement

The animal study was reviewed and approved by Ethics Statement. All experimental procedures were approved by Embrapa Beef Cattle's Ethics Committee on Animal Use according to Protocol 008/2014; coordinated by Vanessa Felipe Souza. e-mail: vanessa.felipe@embrapa.br.

## Author Contributions

RA and RC conceived and designed the study. MG and RC were responsible for the tick rearing, cattle artificial infestation, tick collection, and RNA isolation. PG and AN performed the data analysis. PG, AN, and RA wrote the manuscript. JF critically revised the manuscript. All authors edited and approved the final manuscript.

### Conflict of Interest

The authors declare that the research was conducted in the absence of any commercial or financial relationships that could be construed as a potential conflict of interest.
