# Supplementary figures and images for: Gene Expression in the Salivary Gland of Rhipicephalus (Boophilus) microplus Fed on Tick-Susceptible and Tick-Resistant Hosts
Source: Front Cell Infect Microbiol. 2020 Jan 21;9:477. doi: 10.3389/fcimb.2019.00477 (PMC6985549; doi:10.3389/fcimb.2019.00477)

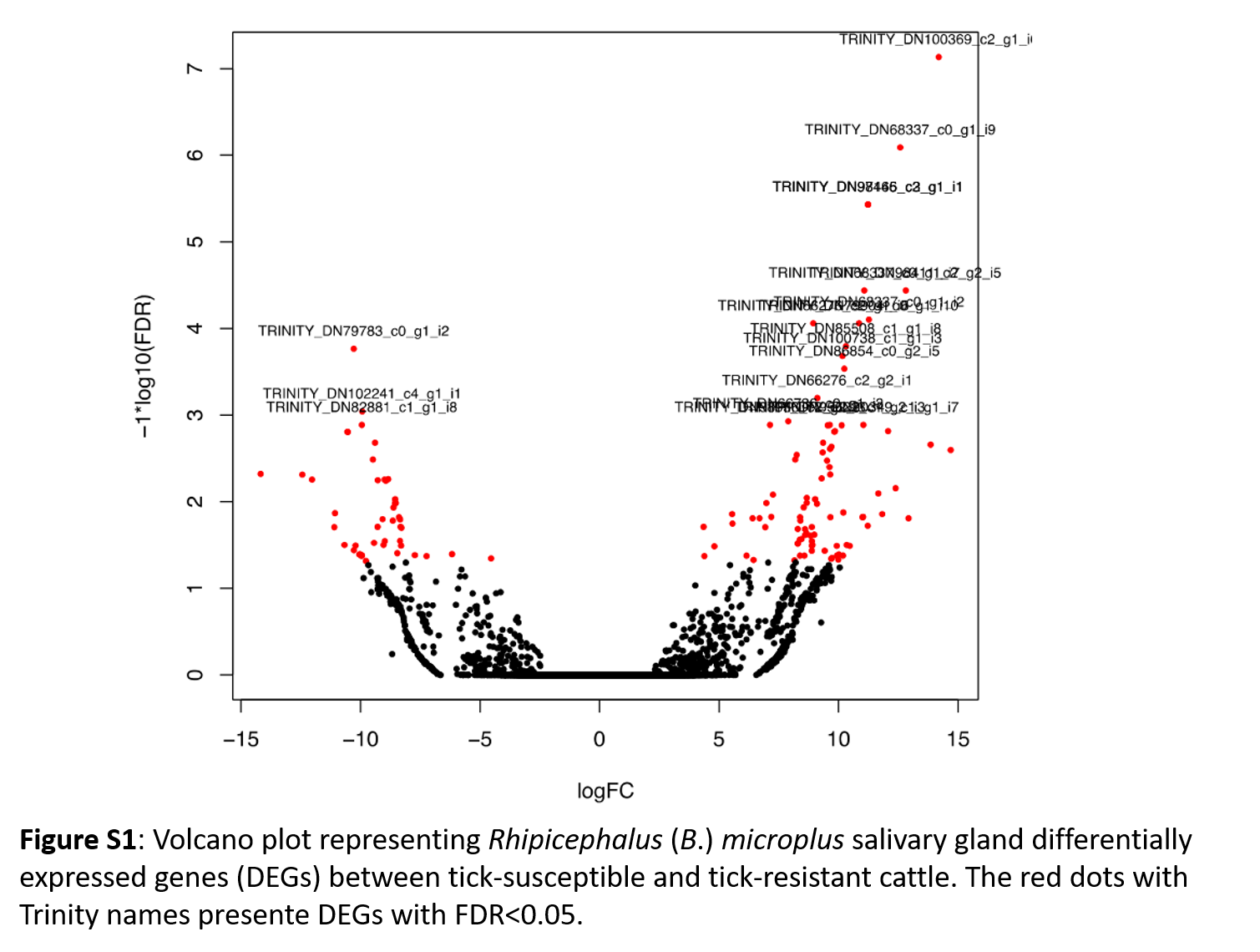

Supplement: Supplementary file 3 [file Image_1.tiff]
